# Clinical Features and Outcomes of IPF Patients Hospitalized for Pulmonary Infection: A Japanese Cohort Study

**DOI:** 10.1371/journal.pone.0168164

**Published:** 2016-12-13

**Authors:** Ryo Yamazaki, Osamu Nishiyama, Hiroyuki Sano, Takashi Iwanaga, Yuji Higashimoto, Hiroaki Kume, Yuji Tohda

**Affiliations:** Department of Respiratory Medicine and Allergology, Kindai University, Faculty of Medicine, Osakasayama, Osaka, Japan; Imperial College London, UNITED KINGDOM

## Abstract

Many patients with idiopathic pulmonary fibrosis (IPF) undergo hospitalizations due to pulmonary infections. We retrospectively investigated the characteristics of hospitalizations due to pulmonary infection in patients with IPF to elucidate causative pathogens and mortality. We reviewed patients with IPF who were admitted between January 2008 and December 2014 for pulmonary infections including pneumonia and bronchitis. The causative pathogen, the relationship between the site of pneumonia and existing IPF radiological patterns on high-resolution chest CT, and predictors of mortality were evaluated. Forty-eight IPF patients were hospitalized a totally of 81 times due to pulmonary infection during the study period. In the 48 first-time admissions after IPF diagnosis, causative pathogens were detected in 20 patients (41.6%). The most common pathogen was *Haemophilus influenzae* (14.5%) followed by *Pseudomonas aeruginosa* (4.1%), *Staphylococcus aureus* (4.1%), *Branhamella catarrhalis* (4.1%), and *Klebsiella pneumoniae* (4.1%). Among all 81 admissions, the most common pathogen was *P*. *aeruginosa* (12.3%), followed by *H*. *influenzae* (8.6%), *S*. *aureus* (6.1%) and *Escherichia coli* (4.9%). No relationship was observed between the detected pathogen and the site of pneumonia. The 30-day and hospital mortality rates were 14.5% and 18.7%, respectively. Pneumonia severity index on admission was significantly associated with both 30-day and hospital mortality. In conclusion, IPF patients hospitalized for pulmonary infections had high 30-day and hospital mortality. In contrast to community-acquired pneumonia, the causative pathogens mainly consisted of gram-negative bacteria. The PSI score may be a significant predictor of mortality. These results provide information for empiric antibiotic selection when treating IPF patients with pulmonary infections.

## Introduction

Idiopathic pulmonary fibrosis (IPF) is a chronic and progressive lung disease that is characterized by the histopathologic pattern of usual interstitial pneumonia [1]. The prognosis is poor and the median survival after diagnosis is 2 to 3 years, although the natural history is variable [1,2].

Many patients with IPF undergo acute respiratory events [3]. Acute respiratory events occur most frequently due to acute exacerbations, followed by pulmonary infections such as pneumonia and bronchitis [3]. Patients with IPF are usually hospitalized when an acute respiratory event occurs, although this depends on regional medical resources. Respiratory-related hospitalizations have recently been recognized as an important factor in IPF outcomes, not only because mortality during respiratory hospitalization is high but also because hospitalization influences subsequent survival [4].

Pulmonary infection is common in patients with IPF and is a major cause of respiratory hospitalization. However, the characteristics of pulmonary infection in IPF have not been fully elucidated. In this study, we retrospectively investigated respiratory hospitalizations in patients with IPF to determine the most common causative pathogens. We hypothesized that the causative pathogens in IPF are different from those in normal community-acquired pneumonia. We also investigated factors that predict mortality in this patient population.

## Materials and Methods

### Patients

We retrospectively examined the medical charts of all patients with IPF who were admitted to the Kindai University Hospital between January 2008 and December 2014 for pulmonary infections, including pneumonia and bronchitis. When the same patient was admitted more than once during the study period, all admissions were included in the analysis. However, admissions occurring less than 2 weeks after the preceding admission were recognized as 1 continuous admission

The diagnosis of IPF was made according to the 2011 ATS/ERS/JRS/ALAT guidelines [1]. Pneumonia was defined as newly developed radiological consolidations or specific local ground-glass opacities on chest radiograph or chest high-resolution computed tomography (HRCT) in a patient with at least 1 of the following: fever, productive cough, or abnormal white blood cell count. If the chest radiograph and/or chest HRCT was unchanged in a symptomatic patient, the patient was diagnosed with bronchitis. Patients with other causes for chest radiographic abnormalities, such as acute exacerbation of IPF, congestive heart failure, pulmonary embolism, or malignancy, were excluded from the study. In particular, patients with new bilateral ground-glass opacities that were consistent with an acute exacerbation of IPF were excluded from the study after careful discussions with multiple specialists.

Approval for the study was provided by the ethics committee of the Kindai University Faculty of Medicine (No. 28–070).

### Pulmonary function tests

Pulmonary function tests performed within 1 year prior to admission were used to assess baseline pulmonary function. Pulmonary function tests, including spirometry and single-breath measurements of diffusing capacity for carbon monoxide (DLco) (CHESTAC-8800; Chest, Tokyo, Japan), were performed according to the European Respiratory Society standards [5, 6]. Results were expressed in absolute values and as a percentage of Japanese normal predictive values [7, 8].

### Microbiology evaluation

Two sets of blood cultures, as well as serum and urine samples, were obtained on the day of admission. Sputum, pleural fluid, endotracheal aspirates, and bronchoalveolar lavage samples were obtained if available. Sputum samples were considered of good quality if they had numerous polymorphonuclear cells (>25 in a ×100 microscopic field) and few squamous epithelial cells (<10 in a ×100 microscopic field) [9].

The etiology of pulmonary infection (culture positive infection) was determined according to the following criteria: (1) an organism was isolated from blood or pleural fluid cultures; (2) a positive urinary antigen for *L pneumophila* and *S*. *pneumoniae*; (3) a morphologically compatible organism observed on Gram staining and later confirmed by sputum culture; (4) a 4-fold rise in IgG titer level between paired sera; (5) a quantitative bacterial growth of 10^5^ cfu/mL or greater in tracheobronchial aspirates, 10^3^ cfu/mL or greater in protected specimen brush fluid, or 10^4^ cfu/mL or greater in bronchoalveolar lavage fluid [10–14]. Culture negative infection was defined when a pathogen was not determined [15].

### Evaluation of pulmonary infection severity and preexisting factors

The pneumonia severity index (PSI) was calculated on the day of admission for all patients included in the study. The PSI is a clinical prediction score for determining short-term mortality in patients with community-acquired pneumonia. The index is based on demographic factors (age, sex, and nursing home residence), coexisting illnesses (neoplastic disease, liver disease, congestive heart failure, cerebrovascular disease, and renal disease), physical examination findings (metal status, respiratory rate, systolic blood pressure, temperature, and pulse), laboratory values (arterial pH, blood urea nitrogen, sodium, glucose, hematocrit, and partial pressure of arterial oxygen), and radiographic findings (pleural effusion). The index is calculated by adding the scores for each factor. The PSI can then be used to risk-stratify patients according to 5 risk classes (I-V) [16].

Preexisting factors that might influence the course of pulmonary infection were also evaluated, including history of pneumococcus vaccination and IPF treatment.

### Assessment of the relationship between the site of pneumonia and existing IPF radiological patterns

In patients with pneumonia, the relationship between the site of pneumonia and existing IPF radiological patterns was evaluated on chest HRCT, given the possibility that structural changes of lung parenchyma such as honeycombing and/or traction bronchiectasis may influence the causative pathogens for pneumonia. When the newly developed pneumonia infiltrate was observed completely or partially within the existing IPF radiological pattern, the patient was recognized as having pneumonia in association with the IPF radiological pattern. If not, the patient was recognized as having pneumonia distinct from the IPF radiological pattern. IPF radiological patterns on chest HRCT included reticular opacities, traction bronchiectasis, and honeycombing. Ground-glass opacities and consolidations were also included in the IPF radiological pattern if they were observed on a pre-hospitalization chest HRCT.

### Assessment of survival

Thirty-day and hospital mortality were evaluated. All deaths were confirmed by hospital chart review.

### Statistical analysis

Continuous variables were summarized by mean±SD and categorical variables were expressed by discrete numbers. The χ^2^ test or Fisher’s exact test were used for categorical data, and the Mann-Whitney U-test was used for continuous data to determine differences in clinical, laboratory, and HRCT data between survivors and non-survivors. Univariate and multivariate analyses using logistic regression models were used to determine potential risk factors for 30-day and hospital mortality. In the mortality analyses, the PSI score (I-V) was treated as a continuous variable. A P value less than or equal to 0.05 was considered significant. Analyses were performed using the PASW statistical package version 18 (SPSS Japan Inc., Tokyo, Japan).

## Results

Seventy-five patients with IPF were hospitalized a total of 159 times due to acute respiratory events during the study period. Among these, 48 patients were hospitalized a total of 81 times due to pulmonary infections. The characteristics of all 48 patients upon their first admission following the diagnosis of IPF are shown in Table 1. The group included 41 men (85.4%) and 7 women (14.5%), and the mean age was 73.8±5.9 years old. Pulmonary function tests obtained within one year prior to admission demonstrated a mean FVC of 65.7±22.5% predicted and a mean DLco of 52.3±14.5% predicted, although the DLco data were only available in 20 patients.

**Table 1:** Characteristics of patients during the first admission following IPF diagnosis.

Characteristic		Value
Age, year		73.8 ± 5.9
Sex	Male	41
	Female	7
Pulmonary function tests	FVC, L	2.0 ± 0.6
	FVC, % predicted	65.7 ± 22.5
	FEV_1_, L	1.7 ± 0.5
	FEV_1_, % predicted	71.3 ± 21.9
	FEV_1_/FVC, %	86.5 ± 9.5
	DLco, mL/min/mmHg	7.4 ± 2.3
	DLco, % predicted	52.3 ± 14.5
PSI classes (episode)	I	0
	II	6
	III	22
	IV	16
	V	4
Pneumococcal vaccine	yes	6
	No	42
Treatment at baseline	Corticosteroid	8
	Pirfenidone	6
	Corticosteroid plus cyclosporine	3
	Cyclosporine	1
	Corticosteroid plus pirfenidone	1
	Corticosteroid, cyclosporine, and pirfenidone	1
	None	28
30-day mortality (%)		14.5
Hospital mortality (%)		18.7

Data are shown as number or mean values with standard deviations unless otherwise indicated.

FEV_1_ = forced expiratory volume in one second, FVC = forced vital capacity, DLco = diffusing capacity for carbon monoxide, PSI = pneumonia severity index

n = 48 except for DLco [22].

The PSI score is shown in Table 1. Six patients were classified as class II, 22 as class III, 16 as class IV, and 4 as class V. No patients were classified as class I. Preexisting factors that might influence the course of infection are also shown in Table 1. Six patients (12.5%) had received the pneumococcal vaccine (23-valent polysaccharide vaccine) prior to admission. Twenty patients (41.6%) were undergoing treatment for IPF. Thirty-day and hospital mortality rates were 14.5% and 18.7%, respectively.

Among the 48 patients admitted for the first time after the diagnosis of IPF, 46 (95.8%) underwent chest HRCT at the time of admission. Among the 81 total admissions, 76 (93.8%) included chest HRCT during the admission. Forty patients were diagnosed with pneumonia during their first-time admission, and 8 patients were diagnosed with bronchitis. Among the total admissions, 69 patients were diagnosed with pneumonia and 12 were diagnosed with bronchitis. Pathogens detected are shown in Table 2. Among the 48 first-time admissions, causative pathogens were detected in 20 patients (41.6%). The most common pathogen detected was *Haemophilus influenzae* (14.5%) followed by *Pseudomonas aeruginosa* (4.1%), *Staphylococcus aureus* (4.1%), *Branhamella catarrhalis* (4.1%), and *Klebsiella pneumoniae* (4.1%). Among the 81 total admissions, the most common pathogen was *P*. *aeruginosa* (12.3%), followed by *H*. *influenzae* (8.6%), *S*. *aureus* (6.1%) and *Escherichia coli* (4.9%).

**Table 2:** Bacteria causing pulmonary infections in patients with IPF.

Pathogens	1st admission N = 48		Total admissions N = 81	
	N	%	N	%
*Pseudomonas aeruginosa*	2	4.1	10	12.3
*Haemophilus influenzae*	7	14.5	7	8.6
*Staphylococcus aureus*	2	4.1	5	6.1
*Escherichia coli*	1	2.0	4	4.9
*Branhamella catarrhalis*	2	4.1	3	3.7
*Klebsiella pneumoniae*	2	4.1	2	2.4
*Streptococcus pneumoniae*	1	2.0	2	2.4
*Serratia marcescens*	1	2.0	1	1.2
*Raoultella planticola*	1	2.0	1	1.2
*Klebsiella oxytoca*	0	0	1	1.2
*Corynebacterium* spp.	1	2.0	1	1.2
Culture negative	28	58.3	45	55.5

One patient who was infected with *Pseudomonas aeruginosa* in the distinct-from-IPF-radiological-pattern group had a mixed infection with *Branhamella catarrhalis* and *Raoultella planticola*.

The mean duration between the day of discharge from the first admission and the day of the second admission was 445±546 days. The mean durations between the second and third admission and between the third and fourth admission were 162±142 days and 274±200 days, respectively.

The relationship between pneumonia location and existing IPF radiological pattern on chest HRCT is shown in Table 3. The pneumonia infiltrate was observed in association with an existing IPF radiological pattern in 29 first-time admission patients (72.5%) and was distinct from existing IPF radiological patterns in 11 patients (27.5%). Among the total admissions, pneumonia infiltrates were observed in association with existing IPF radiological patterns in 52 patients (69.3%), and were distinct from existing IPF radiological patterns in 23 patients (30.6%). There were no statistically significant differences in pathogens isolated from pneumonias associated with existing IPF radiological patterns versus those isolated from pneumonias distinct from IPF radiological patterns (data not shown).

**Table 3:** Site of pneumonia on HRCT.

Pathogens	1st admission		Total admissions	
	Associated	Distinct	Associated	Distinct
	N = 29	N = 11	N = 52	N = 23
*Pseudomonas aeruginosa*	1	1	6	6
*Haemophilus influenzae*	6	1	6	1
*Staphylococcus aureus*	1	1	4	1
*Escherichia coli*	1	0	4	0
*Branhamella catarrhalis*	2	0	2	1
*Klebsiella pneumoniae*	0	1	0	2
*Raoultella planticola*	0	0	0	1
*Streptococcus pneumoniae*	1	0	2	0
*Serratia marcescens*	1	0	1	2
*Klebsiella oxytoca*	0	0	0	1
*Corynebacterium* spp.	1	0	1	0
Culture negative	15	7	26	11

One patient who was infected with *Pseudomonas aeruginosa* in the distinct-from-IPF-radiological-pattern group had a mixed infection with *Branhamella catarrhalis* and *Raoultella planticola*.

The 30-day and hospital mortality rates of patients with IPF included in the present study were 14.5% and 18.7%, respectively. In the survival analyses, univariate logistic regression analysis identified only high PSI (odds ratio (OR) 4.92, 95% confidence interval (CI) 1.36–17.8, *P* = 0.01) as significantly associated with 30-day mortality (Table 4). On the other hand, high PSI (OR 2.77, 95% CI 1.004–7.63, *P* = 0.04) and positive culture (OR 7.14, 95% CI 1.28–50.0, *P* = 0.02) were independently associated with hospital mortality (Table 5). No significant predictors of 30-day or hospital mortality were identified in multivariate logistic regression analyses.

**Table 4:** Univariate logistic regression analysis for 30-day mortality after first admission following IPF diagnosis.

Variable	Odds ratio	95% CI	*P* value
Age	1.11	0.96–1.28	0.15
Male sex	0.34	0.05–2.29	0.27
FVC, L	0.24	0.29–4.25	0.36
FVC, % predicted	0.96	0.89–1.04	0.38
PSI	4.92	1.36–17.8	0.01
PaO_2_ /FiO_2_ ratio	0.99	0.98–1.00	0.13
Steroids with/without Cytotoxic agents, No	0.68	0.17–6.09	0.97
Culture positive	5.88	0.74–33.3	0.10

CI = confidence interval, FiO_2_ = fraction of inspiratory oxygen, FVC = forced vital capacity, PaO_2_ = partial arterial pressure of oxygen, PSI = pneumonia severity index

**Table 5:** Univariate logistic regression analysis for hospital mortality during first admission following IPF diagnosis.

Variable	Odds ratio	95% CI	*P* value
Age	1.14	0.99–1.32	0.05
Male sex	0.51	0.08–3.21	0.47
FVC, L	0.30	0.03–2.68	0.28
FVC, % predicted	0.96	0.89–1.03	0.27
PSI	2.77	1.004–7.63	0.04
PaO_2_ /FiO_2_ ratio	0.99	0.98–1.00	0.05
Steroids with/without Cytotoxic agents, No	1.68	0.28–8.62	0.61
Culture positive	7.14	1.28–50.0	0.02

CI = confidence interval, FiO_2_ = fraction of inspiratory oxygen, FVC = forced vital capacity, PaO_2_ = partial arterial pressure of oxygen, PSI = pneumonia severity index

## Discussion

Many patients with IPF undergo acute respiratory events [3], which, depending on the severity of deterioration and regional medical resources, often leads to hospitalization. Respiratory hospitalization in patients with IPF is associated with high in-hospital mortality and decreased survival [4]. Pulmonary infection is the second leading cause of acute events of IPF [3], hence, knowing the characteristics of respiratory hospitalization in patients with IPF may help determine empiric treatment and improve survival. We thus conducted a retrospective observational study to determine the characteristics of respiratory hospitalizations in this patient population.

Over the 7-year study period, 48 patients with IPF were admitted a total of 81 times for pulmonary infections. Of these, 48 hospitalizations occurred for the first time after the diagnosis of IPF (“first admission”). Our results indicate that pulmonary infection is a significant burden for patients with IPF. During the first admission, the most common pathogen was *H*. *influenzae*, followed by *P*. *aeruginosa*, *S*. *aureus*, *B*. *catarrhalis*, and *K*. *pneumoniae*. Among all admissions, the most common pathogen was *P*. *aeruginosa*, followed by *H*. *influenzae*, *S*. *aureus* and *E*. *coli*. In a survival analysis, high PSI on admission was independently associated with 30-day mortality. In the univariate logistic regression analysis, high PSI on admission and positive cultures were independently associated with hospital mortality. No variable was significantly associated with 30-day or hospital mortality in the multivariate analyses.

We found that *H*. *influenzae* was the most common causative pathogen of pulmonary infection in first time admissions after IPF diagnosis. In contrast, *Streptococcus pneumoniae* is the most frequent cause of community-acquired pneumonia, accounting for 23~35% of cases [10, 17]. In the present study, *S*. *pneumoniae* was observed in only 1 patient (5.0%). Given that only 12.2% of study participants had received pneumococcal vaccine before admission, the low frequency of *S*. *pneumonia* in the present study does not seem to be an effect of vaccination. Song et al. reported on 163 IPF patients with rapid deterioration requiring hospitalization. Among these patients, 51 were diagnosed with pulmonary infections and the most common detected pathogen was *Cytomegalovirus*, followed by *H*. *influenzae* [3]. It is possible that *Cytomegalovirus* was not detected in the present study because viral testing was not routinely performed. However, similar to Song’s study, we found that *H*. *influenzae* was the most frequently detected bacteria. These findings suggest that *H*. *influenzae* infection is a common cause of respiratory hospitalization in patients with IPF, whereas *S*. *pneumoniae* infection is less common in this patient population. We found a particularly high frequency of gram-negative bacterial infections, including *H*. *influenza*, *P*. *aeruginosa*, and *K*. *pneumonia*. The importance of the lung microbiome in IPF has recently been reported in several studies [18, 19]. Molyneaux et al. reported that a 3.4-fold increase in *Haemophilus* sp. was detected in bronchoalveolar lavage fluid from patients with IPF compared to control subjects [18]. The relationship between pulmonary pathogens and the normal microbiome is a rich field of study for further investigation.

Among all admissions, *P*. *aeruginosa* was the most frequently isolated pathogen, and was found in 23% of patients in whom a causative pathogen was detected. There are several possible reasons for this finding. Pneumonia due to *P*. *aeruginosa* frequently occurs in patients with structural lung abnormalities, such as cystic fibrosis or bronchiectasis [20]. Structural lung changes in IPF, including traction bronchiectasis and honeycombing, might influence the types of causative pathogens. However, we found no statistically significant differences in the causative pathogens, including *P*. *aeruginosa*, between pneumonias associated with existing IPF radiological patterns and those that were distinct from existing IPF radiological patterns. *P*. *aeruginosa* pneumonia has been shown to occur frequently in immunocompromised individuals and in patients with recent antibiotic use [20, 21]. Hence, immunosuppressant treatments for IPF and/or antibiotic therapy before hospitalization might have led to our observation of frequent *P*. *aeruginosa* pneumonia in patients with IPF. Specifically, we found that *P*. *aeruginosa* was the most frequently isolated pathogen in patients who were hospitalized several times for pulmonary infections. Another possibility is that a failure to clear the organism during the first admission led to a second admission, particularly in patients who were admitted more than twice within a short interval. It would be necessary to conduct a genetic analysis of the causative pathogens to evaluate this possibility. However, to our knowledge, this is the first study to clarify the characteristics of respiratory hospitalizations in both first-time and repeat admissions in IPF patients.

We found that the 30-day and hospital mortality rates for pulmonary infection in patients with IPF were 14.5% and 18.7%, respectively. This is approximately twice the mortality rate of community-acquired pneumonia requiring hospitalization, which has been reported as 2%~8% [14, 22]. Possible explanations for this high mortality include worsening of IPF itself and the high frequency of gram-negative bacteria as causative pathogens. It is possible that triggered acute IPF exacerbations occurred following a preceding pulmonary infection, although we tried to exclude patients with acute IPF exacerbations from the study. The PSI score may be a predictor of mortality in IPF patients hospitalized for pulmonary infections, although no variables were significantly associated with hospital or 30-day mortality in the multivariate analyses.

Respiratory hospitalization in patients with IPF is associated with high in-hospital mortality and decreased survival following discharge. [4]. Furthermore, respiratory hospitalization can occur independent of a decline in pulmonary function [23]. Therefore, respiratory hospitalization should be recognized as an important outcome measure, not only in clinical trials but also in clinical practice. Respiratory hospitalizations in this patient population are often due to pulmonary infections, hence, understanding the characteristics of pulmonary infections and improving treatment based on these findings may lead to improved survival in patients with IPF.

Our study had several limitations. First, the study was performed at a single center and consisted of a small sample size. The types of pathogens detected may be specific to our local area. Therefore, the results may not be applicable to other patient populations. Second, the study was conducted in a retrospective fashion. Third, a PCR analysis was not performed to detect causative pathogens. Viruses, anaerobes, and some atypical pathogens such as *Mycoplasma pneumoniae* might have been overlooked. Larger, prospective studies are required to confirm our results. Finally, we cannot completely exclude the possibility that patients with acute exacerbation of IPF were included in the study. We tried to recruit only those patients with pure pulmonary infections. However, radiological findings of acute exacerbation sometimes resemble those of pneumonia. Furthermore, acute exacerbation can be triggered by pulmonary infection [24]. The association between pulmonary infection and acute exacerbation of IPF requires further investigation

In conclusion, we found that gram-negative bacteria are the most common pathogens isolated from patients with IPF who are hospitalized for pulmonary infections. This is in contrast to the pathogens commonly isolated from patients with community-acquired pneumonia, which tend to be gram-positive. The PSI score may be a significant predictor of mortality. Our results may provide guidance in selecting antibiotics when treating IPF patients with pulmonary infections.
